# Prenatal Maternal Occupation and Child Epigenetic Age Acceleration in an Agricultural Region: NIMHD Social Epigenomics Program

**DOI:** 10.1001/jamanetworkopen.2024.21824

**Published:** 2024-07-29

**Authors:** Saher Daredia, Anne K. Bozack, Corinne A. Riddell, Robert Gunier, Kim G. Harley, Asa Bradman, Brenda Eskenazi, Nina Holland, Julianna Deardorff, Andres Cardenas

**Affiliations:** 1 Division of Epidemiology, School of Public Health, University of California, Berkeley; 2 Department of Epidemiology and Population Health, School of Medicine, Stanford University, Stanford, California; 3 Division of Biostatistics, School of Public Health, University of California, Berkeley; 4 Center for Environmental Research and Community Health, School of Public Health, University of California, Berkeley; 5 Division of Community Health Sciences, School of Public Health, University of California, Berkeley; 6 Department of Public Health, University of California, Merced; 7 Division of Environmental Health Sciences, School of Public Health, University of California, Berkeley; 8 Department of Pediatrics, School of Medicine, Stanford University, Stanford, California

## Abstract

**Importance:**

Research on fetal epigenetic programming suggests that the intrauterine environment can have long-term effects on offspring disease susceptibility.

**Objective:**

To examine the association between prenatal maternal occupation and child epigenetic age acceleration (EAA) among a farmworker community.

**Design, Setting, and Participants:**

This cohort study included participants in the Center for the Health Assessment of Mothers and Children of Salinas, a prospective, Latino, prebirth cohort. Pregnant women were recruited from October 1, 1999, to October 1, 2000, from 6 community clinics in California’s Salinas Valley agricultural region. Participants were 18 years or older, English or Spanish speaking, Medicaid eligible, and at 20 weeks’ gestation or earlier at enrollment. Mother-child pairs who had blood DNA methylation measured at the ages of 7, 9, and 14 years were included. Data were analyzed from July 2021 to November 2023.

**Exposures:**

Prenatal maternal occupation was ascertained through study interviews conducted during prenatal visits and shortly after delivery.

**Main Outcomes and Measures:**

Child EAA at 7, 9, and 14 years of age was estimated using DNA methylation–based epigenetic age biomarkers. Three EAA measures were calculated: the Horvath EAA, skin and blood EAA, and intrinsic EAA. Linear mixed-effects models were used to estimate longitudinal associations of prenatal maternal occupation and child EAA, adjusting for confounders and prenatal organophosphate pesticide exposure.

**Results:**

Analyses included 290 mother-child pairs (mean [SD] maternal age at delivery, 26.5 [5.2] years; 152 [52.4%] female infants); 254 mothers (87.6%) were born in Mexico, 33 (11.4%) in the US, and 3 (1.0%) in other countries; and 179 families (61.7%) were below the federal poverty line during pregnancy. Mothers reported engaging in several types of work during pregnancy, including agricultural fieldwork (90 [31.0%]), other agricultural work (40 [13.8%]), nonagricultural work (53 [18.3%]), or no work (107 [36.9%]). Children whose mothers worked in agricultural fields during pregnancy had a mean of 0.66 (95% CI, 0.17-1.15) years of greater Horvath EAA, 0.62 (95% CI, 0.31-0.94) years of greater skin and blood EAA, and 0.45 (95% CI, 0.07-0.83) years of greater intrinsic EAA compared with children whose mothers did not work during pregnancy.

**Conclusions and Relevance:**

In this cohort study, prenatal maternal agricultural fieldwork was associated with accelerated childhood epigenetic aging independent of organophosphate pesticide exposure. Future research on which factors related to agricultural fieldwork accelerate aging in the next generation can inform targeted prevention programs and policies that protect children’s health.

## Key Points

**Question:**

Is prenatal maternal occupation associated with child epigenetic aging in a farmworker population?

**Findings:**

In this cohort study of 290 mother-child pairs, children whose mothers engaged in agricultural fieldwork during pregnancy had greater epigenetic age acceleration as measured by several DNA methylation–based biomarkers compared with those whose mothers did not work during pregnancy. These associations were independent of sociodemographic characteristics and prenatal pesticide exposure.

**Meaning:**

These findings suggest that children of agricultural fieldworkers, a vastly understudied population, may experience increased rates of biological aging early in life and greater risk of age-related diseases later in life due to prenatal stressors.

## Teaser

This cohort study of mother-infant pairs examines the association between prenatal maternal occupation and epigenetic aging among children in a Latino agricultural community in California.

## Introduction

Evidence suggests that exposures in utero can become biologically embedded via epigenetic mechanisms, affecting fetal development and disease onset later in life.^1,2^ Genome-wide changes in DNA methylation (DNAm), a type of epigenetic modification, are strongly correlated with aging.^3,4^ Gene-specific DNAm from multiple human tissue types has been leveraged to develop biomarkers of biological aging, known as epigenetic clocks. Epigenetic age acceleration (EAA), which is the difference between epigenetic age as estimated by these clocks and chronological age, is closely associated with morbidity and mortality in adults.^5,6,7^ However, recent studies demonstrate that epigenetic aging processes begin as early as conception,^8,9^ emphasizing the need to consider prenatal influences on aging.

Although some environmental and social exposures during pregnancy affect epigenetic age measured at birth,^8,9,10,11,12,13,14,15,16,17^ data on whether the prenatal environment affects epigenetic aging throughout childhood are limited. Prenatal smoking, gestational diabetes, and exposure to phthalates have been associated with altered epigenetic aging in early to middle childhood.^9,18,19,20^ However, only a small number of studies have examined prenatal exposures and prospective measurements of child EAA,^21,22,23^ and even fewer have followed up youth into adolescence.^21^ Moreover, no epigenetic studies have focused on maternal occupation during pregnancy, which is an important area to consider when assessing pregnancy-related stress and burden. Prenatal maternal stress alters DNAm signatures and downstream gene expression among newborns.^24,25,26^ Longitudinal studies are needed to understand the persistence of these epigenetic modifications in childhood and beyond,^27^ especially among low-income populations and mothers from underrepresented backgrounds, who are disproportionately exposed to occupational stressors during pregnancy that might affect their children.^28^

To address these research gaps, we tested the association between prenatal maternal occupation and epigenetic aging among children in a Latino agricultural community. Pregnant farmworkers, especially those working in agricultural fields, are particularly vulnerable to occupational risk factors, including pesticide exposure, heat stress, and physical exertion.^29,30^ Beyond workplace hazards, farmworker families also often experience food and housing insecurity, fears related to immigration status, cultural barriers, and inadequate access to medical and social services.^31,32,33,34^ Given these stressors, we hypothesized that prenatal maternal agricultural work is associated with accelerated epigenetic aging in childhood.

## Methods

### Study Population

This cohort study used data from the Center for the Health Assessment of Mothers and Children of Salinas (CHAMACOS), a longitudinal, prebirth cohort composed of primarily Mexican American children born in California’s agricultural Salinas Valley. Eligible pregnant women (≥18 years of age, English or Spanish speaking, ≤20 weeks’ gestation at enrollment, Medicaid eligible, and planning to deliver at the county hospital) were recruited between October 1, 1999, and October 1, 2000, from 6 community clinics, as described elsewhere.^35,36^ Of 601 initial enrollees, 526 (87.5%) were followed up to the delivery of live, singleton newborns. The study continued to follow up mother-child pairs after delivery. A phlebotomist collected child blood samples via venipuncture at study visits conducted when the children were 7, 9, and 14 years old. We restricted these analyses to 290 mother-child pairs who reported prenatal maternal occupation, had available child chronological age data (estimated to the day of the study visit), and provided blood samples during at least 1 study visit: 7 (n = 182), 9 (n = 239), and 14 (n = 185) years of age. Details on the number of repeated measures per participant and overlapping participants between time points are presented in eTables 1 and 2 in Supplement 1. The University of California, Berkeley Committee for the Protection of Human Subjects approved all study activities. Written informed consent was obtained from mothers. Child verbal assent was obtained from children aged 7 and 9 years; written assent was obtained from children aged 14 years. This study followed the Strengthening the Reporting of Observational Studies in Epidemiology (STROBE) reporting guideline for cohort studies.

### DNAm and Epigenetic Aging Measures

DNA methylation was measured from blood samples of children aged 9 years (Illumina Infinium HumanMethylation450 BeadChip; Illumina Inc) and from blood samples of children aged 7 and 14 years with the EPIC BeadChip (Illumina Inc), according to the manufacturer’s protocol.^37,38^ DNA methylation profiling and quality control are described in the eMethods in Supplement 1.

Epigenetic age measures from 6 clocks (Horvath pan-tissue,^39^ skin and blood,^40^ Hannum,^41^ PhenoAge,^42^ DNAmTL,^43^ and GrimAge^44^) were estimated from the DNAm data at each time point using 3 publicly available methods: (1) the methylCIPHER R package,^45^ (2) the online Clock Foundation calculator,^39^ and (3) a principal component–based estimation.^46^ The performance of each clock and its estimation method was evaluated by Pearson correlation coefficients (*r*) and median absolute error (MAE) between epigenetic age and chronological age (eTable 3 in Supplement 1). The estimation method producing the highest Pearson *r* followed by the lowest MAE was systematically chosen for each clock for statistical analyses (eFigure 1 in Supplement 1). In primary analyses, epigenetic age from the Horvath pan-tissue clock (referred to here as the Horvath clock) and skin and blood clock were selected due to goodness of fit with chronological age in our sample (*r* ≥ 0.8, MAE ≤ 2 years), original training data including pediatric populations, and applicability throughout the human lifespan.^39,40^ Secondary analyses were conducted using the other epigenetic aging biomarkers (Hannum, PhenoAge, DNAmTL, and GrimAge).

Epigenetic age acceleration was calculated for each clock as the residuals from a linear regression of epigenetic age on chronological age. We used Horvath EAA, skin and blood EAA, and intrinsic EAA (IEAA) as the primary outcomes. Intrinsic EAA is based on the Horvath clock but independent of changes in blood cell type composition and indicative of cell-intrinsic aging.^47^ Details on how IEAA was calculated are available in the eMethods in Supplement 1.

### Maternal Occupation During Pregnancy

Trained bilingual staff members interviewed pregnant mothers at a median (IQR) of 13 (10-17) weeks’ and 26 (25-27) weeks’ gestation and 1 to 7 days after delivery. During each interview, mothers were asked if they were currently working and, if so, whether they had engaged in specific tasks (yes or no) at each of their jobs, if multiple. Each job was classified as agricultural fieldwork, other general agricultural work, or nonagricultural work. Agricultural fieldwork included harvesting, thinning, or weeding crops. Other agricultural work included applying and handling fertilizers, handling pesticides, operating equipment or tractors, serving as foreperson, or working in a packing shed, nursery, or greenhouse.

If mothers reported working in the fields during pregnancy and not participating in other agricultural tasks, their occupation was classified as agricultural fieldwork. Maternal occupation was categorized as other agricultural work if agricultural tasks other than fieldwork were reported during any study interview. Nonagricultural work was assigned if mothers reported working, but never in agricultural settings. Mothers were categorized as not having worked during their pregnancy if they reported not having a job during all 3 interviews. During interviews, working mothers also self-reported the physical difficulty of their jobs (not at all, not very, somewhat, or very strenuous) and the average hours per day spent standing on their feet and stooping or bending at work.

### Covariates

#### Mother-Child Sociodemographic Characteristics

Covariates were selected a priori using a directed acyclic graph^48^ and included maternal age at delivery, prepregnancy body mass index, maternal educational level (6th grade or less, 7th-12th grade, high school graduate or more), marital status (married, living as married, separated, divorced, or single), parity (nulliparous or multiparous), prenatal smoking and alcohol consumption (no or yes), poverty status during pregnancy (poverty line or below, between the poverty line and 200%, or higher than 200% of the poverty line as determined by US Census Bureau thresholds), and child sex.

#### Prenatal Organophosphate Pesticide Exposure

From 1999 to 2000, the prenatal period for children in the CHAMACOS study, nearly a half-million pounds of organophosphate pesticides were applied in Salinas Valley.^49^ In this study, we assessed prenatal organophosphate pesticide exposure using 2 methods. Dialkylphosphate metabolites, a proxy of exposure to organophosphate pesticides, were measured from maternal urine samples collected during the 2 prenatal study interviews, as described elsewhere.^50^ Metabolite levels below the limit of detection were randomly imputed based on a log-normal probability distribution.^51^ Urinary dialkylphosphate concentrations were averaged across both samples. Using California Pesticide Use Reporting data from 1999 to 2001, we also estimated kilograms of organophosphate pesticides applied within 1 km of each mother’s residence from estimated conception date to delivery, as described elsewhere.^52,53^

### Statistical Analysis

We described participant characteristics with means (SDs) for continuous measurements and numbers (percentages) for categorical variables. Linear mixed-effects regression models were used to examine associations between prenatal maternal occupation and longitudinal measures of child Horvath EAA, skin and blood EAA, and IEAA. Models included random slopes and intercepts to account for within- and between-child variability in the outcome. Models were adjusted for child chronological age as recommended,^54^ mother-child sociodemographic characteristics, and prenatal organophosphate pesticide exposure. We adjusted for both log_10_-transformed urinary dialkylphosphates and log_2_-transformed California Pesticide Use Reporting estimates because a previous study in our cohort showed that these were not highly correlated and provided complementary measures of organophosphate pesticide exposure.^53^ The threshold for statistical significance was defined using 95% CIs.

As a sensitivity analysis, statistical interaction terms between child age and prenatal maternal occupation were added to the previously described models as recommended.^55^ A likelihood ratio test was used to assess whether model fit was improved by including interaction terms. Additional sensitivity analyses adjusted for (1) maternal years in the US at child’s birth, (2) prenatal paternal occupation, (3) the number of farmworkers living in the household during pregnancy, and (4) replaced maternal occupation with physical exertion at mother’s work during pregnancy as the main exposure. Data were analyzed from July 2021 to November 2023. Analyses were performed using R, version 4.3.1 (R Foundation for Statistical Computing).^56^

## Results

### Participant Characteristics

Among 290 mother-child pairs (mean [SD] maternal age at delivery, 26.5 [5.2] years; 152 female [52.4%] and 138 male [47.6%] infants) included in the analysis, 254 mothers (87.6%) were born in Mexico, 33 (11.4%) in the US, and 3 (1.0%) in other countries (specific countries not reported because of small sample size and possible identification of participants), and 282 (97.2%) self-identified as Mexican or Mexican American (race not reported for the other 2.8% to protect patient anonymity). A total of 179 mothers (61.7%) were living at or below the federal poverty line during pregnancy, with 279 (96.2%) living below 200% of the poverty line. Ninety mothers (31.0%) reported agricultural field work; 40 (13.8%), other agricultural work; 53 (18.3%), nonagricultural work; and 107 (36.9%), no work during pregnancy. The Table describes sociodemographic characteristics for our analytic sample overall and by each time point. eTable 4 in Supplement 1 describes characteristics for our sample by prenatal occupation category. eTable 5 in Supplement 1 shows a comparison of characteristics between included and excluded mother-child pairs. Mothers included in our analyses were slightly older and had lived in the US for a longer duration compared with those excluded.

**Table.** Sociodemographic Characteristics of 290 Mother-Child Pairs Included in the Study at Child Ages of 7, 9, and 14 Years^a^

Characteristic	Total (N = 290)	Age 7 y (n = 182)	Age 9 y (n = 239)	Age 14 y (n = 185)
Maternal characteristics				
Age at delivery, mean (SD), y	26.5 (5.2)	26.1 (5.0)	26.5 (5.2)	26.2 (4.9)
Prepregnancy BMI, mean (SD)	27.4 (5.4)	27.4 (5.4)	27.5 (5.4)	27.6 (5.7)
Highest level of education				
6th Grade or less	128 (44.1)	81 (44.5)	107 (44.8)	79 (42.7)
7th-12th Grade	102 (35.2)	64 (35.2)	84 (35.1)	67 (36.2)
High school or more	60 (20.7)	37 (20.3)	48 (20.1)	39 (21.1)
Marital status				
Married	133 (45.9)	79 (43.4)	110 (46.0)	79 (42.7)
Living as married	105 (36.2)	72 (39.6)	88 (36.8)	76 (41.1)
Separated	11 (3.8)	7 (3.8)	7 (2.9)	7 (3.8)
Divorced	5 (1.7)	3 (1.6)	5 (2.1)	4 (2.2)
Single	35 (12.1)	21 (11.5)	28 (11.7)	19 (10.3)
Missing	1 (0.3)	0	1 (0.4)	0
Parity				
Nulliparous	95 (32.8)	63 (34.6)	74 (31.0)	56 (30.3)
Multiparous	195 (67.2)	119 (65.4)	165 (69.0)	129 (69.7)
Country of origin				
US	33 (11.4)	22 (12.1)	30 (12.6)	25 (13.5)
Mexico	254 (87.6)	158 (86.8)	206 (86.2)	158 (85.4)
Other^b^	3 (1.0)	2 (1.1)	3 (1.3)	2 (1.1)
Self-reported race				
Mexican or Mexican American	282 (97.2)	179 (98.4)	232 (97.1)	182 (98.4)
Other^c^	8 (2.8)	3 (1.6)	7 (2.9)	3 (1.6)
Length of time in US at child’s birth, y				
≤1	52 (17.9)	31 (17.0)	42 (17.6)	30 (16.2)
2-5	81 (27.9)	52 (28.6)	67 (28.0)	52 (28.1)
6-10	86 (29.7)	55 (30.2)	73 (30.5)	54 (29.2)
≥11	45 (15.5)	27 (14.8)	34 (14.2)	31 (16.8)
Entire life	26 (9.0)	17 (9.3)	23 (9.6)	18 (9.7)
Poverty status during pregnancy				
At or below poverty line	179 (61.7)	113 (62.1)	150 (62.8)	113 (61.1)
Between poverty line and 200%	100 (34.5)	64 (35.2)	78 (32.6)	67 (36.2)
>200% Poverty line	11 (3.8)	5 (2.7)	11 (4.6)	5 (2.7)
Smoking during pregnancy				
No	279 (96.2)	174 (95.6)	230 (96.2)	177 (95.7)
Yes	11 (3.8)	8 (4.4)	9 (3.8)	8 (4.3)
Alcohol consumption during pregnancy				
No	221 (76.2)	139 (76.4)	182 (76.2)	139 (75.1)
Yes	67 (23.1)	42 (23.1)	56 (23.4)	46 (24.9)
Missing	2 (0.7)	1 (0.5)	1 (0.4)	0
Occupation during pregnancy				
Agricultural fieldwork	90 (31.0)	59 (32.4)	74 (31.0)	59 (31.9)
Other agricultural work	40 (13.8)	22 (12.1)	35 (14.6)	27 (14.6)
Nonagricultural work	53 (18.3)	37 (20.3)	39 (16.3)	36 (19.5)
Did not work	107 (36.9)	64 (35.2)	91 (38.1)	63 (34.1)
Mean (SD) prenatal urinary dialkylphosphate, nmol/g of creatinine	287.9 (348.7)	278.9 (335.1)	280.9 (347.6)	265.4 (309.1)
Missing, No.	1	0	1	0
Prenatal wind-weighted kilograms of organophosphate pesticides applied within 1 km of residence, mean (SD)	22.5 (34.7)	23.9 (36.7)	20.3 (31.9)	23.8 (38.8)
Missing, No.	1	0	1	0
Child characteristics				
Sex				
Female	152 (52.4)	99 (54.4)	129 (54.0)	97 (52.4)
Male	138 (47.6)	83 (45.6)	110 (46.0)	88 (47.6)
Age at study visit, mean (SD), y	NA	7.1 (0.2)	9.1 (0.2)	14.1 (0.2)
DNAm array	NA	EPIC	450K	EPIC
Horvath EA, y	NA	7.4 (1.7)	8.3 (2.0)	16.4 (3.2)
Horvath EAA, y	NA	0.0 (1.7)	0.0 (2.0)	0.0 (3.2)
Skin and blood EA, y	NA	4.3 (0.9)	7.8 (1.6)	13.1 (1.6)
Skin and blood EAA, y	NA	0.0 (0.8)	0.0 (1.6)	0.0 (1.6)
IEAA, y	NA	0.0 (1.7)	0.0 (1.2)	0.0 (2.2)

Abbreviations: BMI, body mass index (calculated as weight in kilograms divided by height in meters squared); DNAm, DNA methylation; EA, epigenetic age; EAA, epigenetic age acceleration; IEAA, intrinsic epigenetic age acceleration; NA, not applicable.

^a^ Data are presented as number (percentage) of participants unless otherwise indicated.

^b^ Other countries of origin not reported to protect patient anonymity.

^c^ Other self-reported race not reported to protect patient anonymity.

### Performance of Epigenetic Clocks

**Figure 1.**
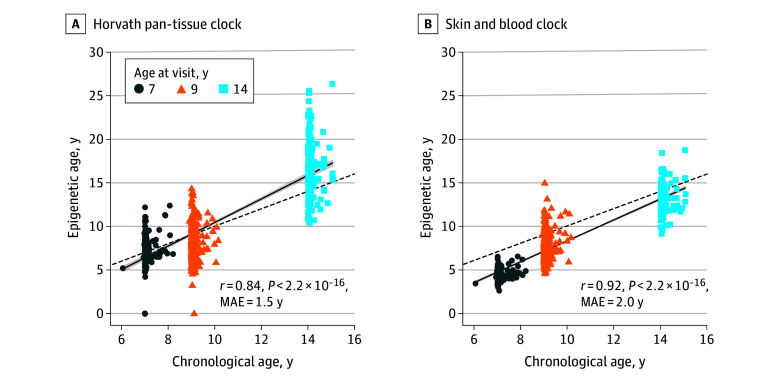
Performance of the Horvath Pan-Tissue and Skin and Blood Epigenetic Clocks in 290 Children Aged 7 to 14 Years From the Center for the Health Assessment of Mothers and Children of Salinas Pearson correlation coefficients (*r*) and median absolute errors (MAEs) between child chronological age based on birth date and epigenetic age were estimated by the Horvath pan-tissue and skin and blood epigenetic clocks. The linear trendline and 95% CIs are plotted as a solid line with shaded area, and the identity line (y = x) is plotted as a dashed line. If chronological and epigenetic age are equivalent for a participant, their datapoint falls exactly on the identity line.

Epigenetic aging measures from the Horvath and skin and blood epigenetic clocks were good estimates of child chronological age across 7, 9, and 14 years as measured by Pearson correlation coefficients (Horvath *r* = 0.84 [MAE = 1.5 years]; skin and blood *r* = 0.92 [MAE = 2.0 years]) (Figure 1). Other clocks performed relatively well in accuracy but had weaker correlations with chronological age and higher MAEs (eFigure 1 in Supplement 1); therefore, Horvath, skin and blood, and IEAA were used in our primary analyses. Correlations between chronological age and epigenetic age estimates by each time point are presented in eFigure 2 in Supplement 1.

### Association Between Prenatal Maternal Occupation and Child EAA

**Figure 2.**
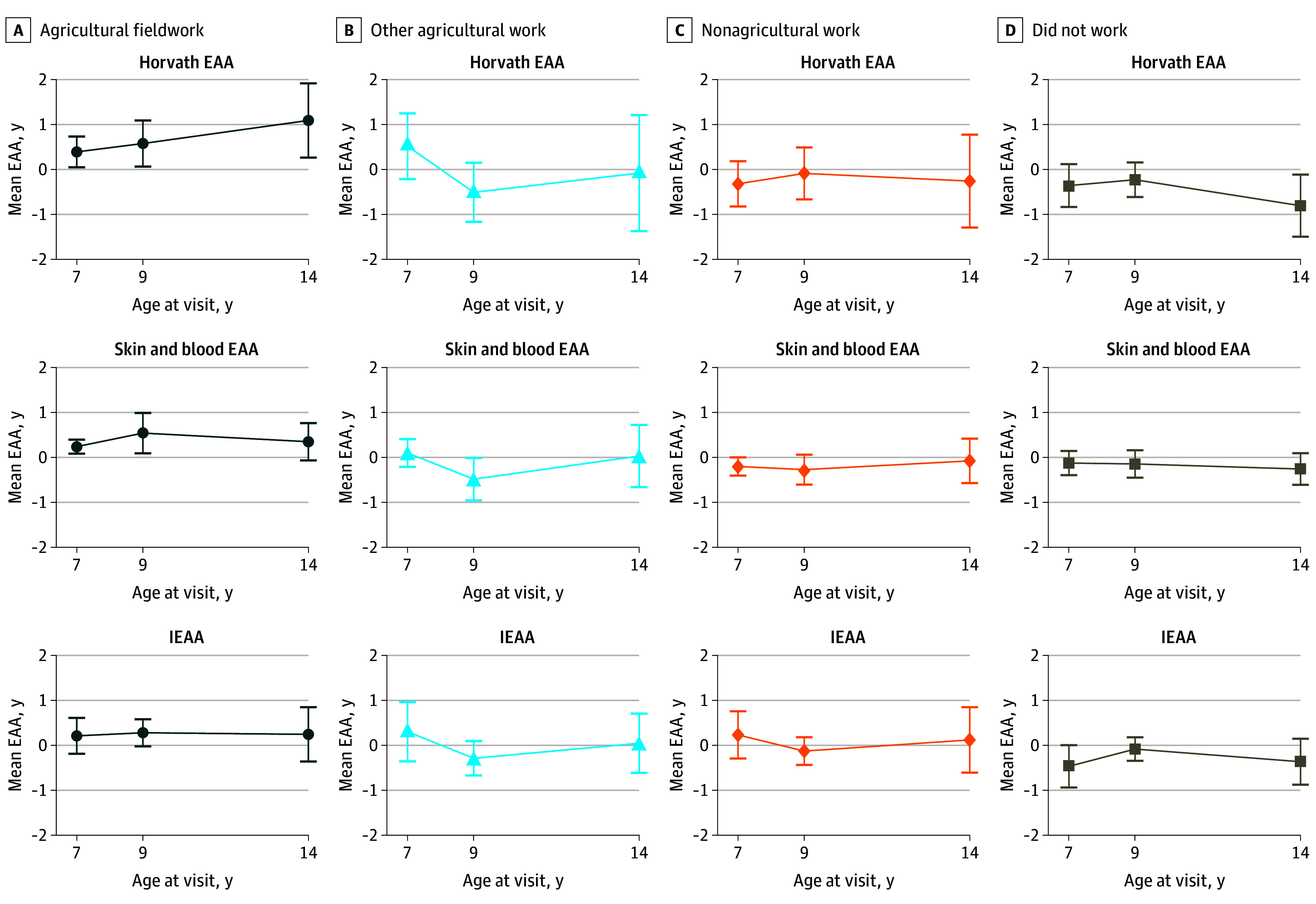
Unadjusted Mean (95% CI) Horvath Epigenetic Age Acceleration (EAA), Skin and Blood EAA, and Intrinsic EAA (IEAA) in Childhood by Prenatal Maternal Occupation Mean EAA in years are given at different time points in childhood for children born to mothers working in different occupations during pregnancy. These results are not adjusted for hypothesized confounders. Deviations from an EAA of 0 years indicate either mean accelerated or decelerated epigenetic aging at the aggregate level.

**Figure 3.**
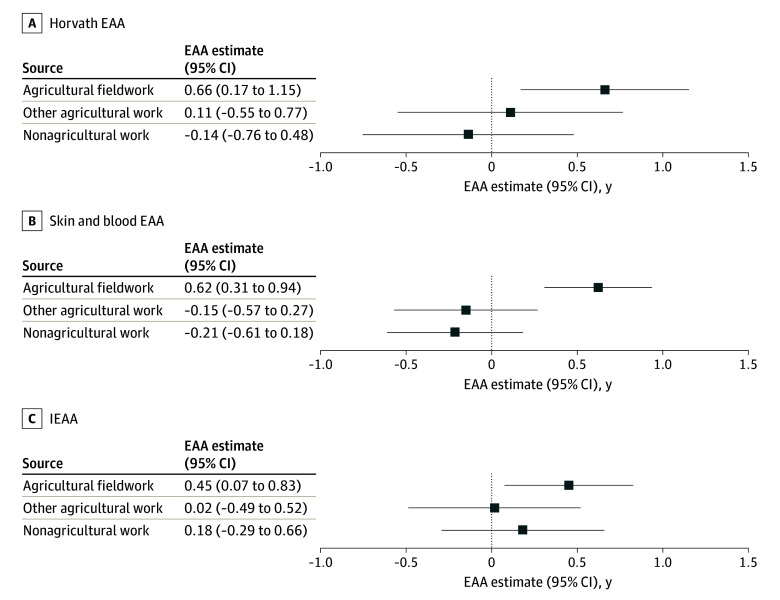
Adjusted Associations Between Prenatal Maternal Occupation and Child Epigenetic Age Acceleration (EAA) Compared With Children Whose Mothers Did Not Work During Pregnancy Regression coefficients in years and 95% CIs (error bars) derived from linear mixed-effects models adjusted for sociodemographic covariates (maternal age at delivery, prepregnancy body mass index, baseline maternal educational level, baseline maternal marital status, parity, poverty status during pregnancy, smoking and alcohol consumption during pregnancy, and child sex) and prenatal organophosphate pesticide exposure (log_10_-transformed mean prenatal urinary dialkylphosphate concentrations and log_2_-transformed kilograms of organophosphate pesticides used within 1 km of the maternal residence during pregnancy). IEAA indicates intrinsic EAA.

Unadjusted mean child Horvath, skin and blood, and IEAA measures were consistently elevated (>0 years) at 7, 9, and 14 years of age among children whose mothers engaged in agricultural fieldwork during pregnancy (Figure 2). In longitudinal adjusted models, children whose mothers were agricultural fieldworkers during pregnancy had a mean of 0.66 (95% CI, 0.17-1.15) years greater Horvath EAA, 0.62 (95% CI, 0.31-0.94) years greater skin and blood EAA, and 0.45 (95% CI, 0.07-0.83) years greater IEAA compared with children whose mothers did not work during pregnancy (Figure 3). Associations between other agricultural work and nonagricultural work during pregnancy with child EAA were consistently close to the null (Figure 3).

In secondary adjusted analyses, prenatal maternal agricultural fieldwork was also associated with greater mean child EAA from the Hannum (1.43 years; 95% CI, 0.34-2.52 years) and PhenoAge (0.74 years; 95% CI, 0.18-1.31 years) clocks and with a negative mean age–adjusted estimate of child DNAmTL (−0.05; 95% CI, −0.09 to −0.01), indicating shorter telomere length—a hallmark of increased biological aging (eFigure 3 in Supplement 1).

### Sensitivity Analyses

Estimates from models with statistical interaction terms provide evidence that the association of prenatal maternal agricultural fieldwork with child Horvath EAA may be greater with increasing child chronological age (eTable 6 in Supplement 1). Compared with children with mothers who did not work during pregnancy, children with mothers who engaged in agricultural fieldwork during pregnancy had a mean Horvath EAA that was 0.38 (95% CI, −0.16 to 0.92) years greater at 7 years of age, 0.70 (95% CI, 0.21-1.18) years greater at 9 years of age, and 1.49 (95% CI, 0.64-2.34) years greater at 14 years of age.

Prenatal maternal agricultural fieldwork remained associated with increased mean child EAA in models that additionally adjusted for (1) mothers’ years in the US as a proxy for social support and immigration-related stressors, (2) prenatal paternal occupation, or (3) the number of farmworkers living in the household during pregnancy. Mothers’ self-reported physical difficulty at work and mean hours per day standing at work were not associated with child EAA. A 1-hour increase in mothers’ mean hours per day stooping or bending at work was associated with increased mean child skin and blood EAA (0.10 years; 95% CI, 0.01-0.18 years) and IEAA (0.11 years; 95% CI, 0.01-0.20 years).

## Discussion

In this study, we tested prospective associations of maternal occupation during pregnancy with epigenetic aging across childhood in the CHAMACOS prebirth cohort. We found that prenatal maternal agricultural fieldwork was associated with accelerated child epigenetic aging independent of sociodemographic characteristics and prenatal organophosphate pesticide exposure. Our findings were consistent across multiple epigenetic clock biomarkers. Each clock was developed using DNAm at different CpG (cytosine-phosphate-guanine) sites, thus capturing different aspects of biological aging. Therefore, our results suggest that prenatal maternal agricultural fieldwork may impact several biological aging processes in children, including those independent of age-related changes in blood cell type composition as reflected by IEAA.

Theoretical frameworks, such as the DOHaD (Developmental Origins of Health and Disease),^57,58^ posit that maternal stressors during pregnancy can cumulatively influence offspring’s health in later life. Mothers in our study population, an immigrant farmworker community, face a unique combination of adverse social and chemical exposures during pregnancy. Agricultural fieldworkers, in particular, are at the bottom of a labor hierarchy on farms that is largely defined by race, class, and citizenship.^59^ Moreover, female agricultural fieldworkers are routinely exposed to sexual harassment in the fields.^60^ Those who are pregnant are particularly vulnerable to harsh working conditions, such as irregular access to restrooms and drinking water, pesticide exposure, and prolonged, physically demanding work in high temperatures,^29^ all of which have been independently linked to adverse pregnancy and birth outcomes.^61,62,63,64^ Exposures to medium- and long-term heat^65^ and organochlorine pesticides^66^ have been associated with greater EAA in adults. Maternal psychosocial stress, including prenatal anxiety and perceived discrimination, have intergenerational consequences on child epigenetic aging.^22,67^ Given these findings, we hypothesize that the accumulation of multiple stressors associated with prenatal maternal agricultural fieldwork, not any one stressor alone, accelerated child epigenetic aging in our cohort.

To our knowledge, this is the first study to assess the association of prenatal maternal occupation with child epigenetic aging. Numerous studies have shown that chemical, psychosocial, and ergonomic hazards in prenatal maternal work environments have downstream deleterious effects on birth outcomes and child health.^68^ Our findings suggest that epigenetic pathways may be involved in these observed associations. Our study also considers a population that is typically underrepresented in research, especially in genomics. Diversifying cutting-edge research on biomarkers of aging will enable us to better understand how the social environment influences deviations in these biomarkers and develop health interventions for vulnerable populations. Moreover, our work provides support for workplace accommodations to ensure the safety of pregnant farmworkers, as well as expanded options for their paid leave during pregnancy.

Additional studies are needed to clarify the long-term health implications of altered aging processes in early life. Although the EAA of multiple clocks in adults is associated with morbidity and mortality, consequences of altered EAA in pediatric populations have not been characterized but provide immense opportunity for disease prevention. A handful of studies have linked EAA and maturation processes, showing that EAA is associated with higher weight for age, taller height for age, and earlier pubertal onset.^69,70,71^ In turn, early pubertal timing is associated with later risk of adult obesity, type 2 diabetes, and cardiovascular disease.^72^ More longitudinal research is needed to evaluate the persistence of epigenetic aging trajectories and whether accelerated epigenetic aging in childhood and adolescence impacts risk of chronic diseases.

### Limitations

Our study has some limitations. Maternal agricultural fieldwork may encompass a variety of exposures, including pesticides, heat, physical exertion, and socioeconomic adversity beyond the workplace, some of which may act as mechanisms on the causal path to epigenetic aging. We found robust associations after controlling for organophosphate pesticide exposure quantified by 2 different exposure assessment methods, as well as immigration-related stressors assessed using maternal years in the US. Although our sensitivity analysis showed positive associations with prenatal occupational bending and stooping and child EAA from some clocks, this measure did not capture physical exertion outside the workplace. Other potential prenatal (eg, mothers’ exposure to heat, harassment at work, and pesticide mixtures) and postnatal (eg, early-life socioeconomic status and pesticide exposure) mechanisms were also not captured in our study. Future research should focus on identifying mediating pathways to inform targeted preventive interventions and policies. In addition, although we adjusted for covariates that are proxies for maternal socioeconomic status and acculturation, there may be residual confounding in our study from unmeasured variables that may have influenced mothers’ choice of occupation (or lack thereof) during pregnancy. Finally, our study sample was largely composed of low-income, immigrant Latino families, which limits generalizability of our results to other populations. Nevertheless, we believe it is crucial to continue expanding social and environmental epigenomics research to more diverse study populations.

## Conclusions

This longitudinal cohort study found that prenatal maternal agricultural fieldwork was associated with child EAA among a Latino prebirth cohort, independent of prenatal organophosphate pesticide exposure and sociodemographic characteristics. Understanding factors that accelerate early-life biological aging in vulnerable populations, such as farmworker communities, may help to identify targets for adult disease prevention and mitigate health disparities.

## Notes

Supplement 1. **eMethods.** Supplemental Methods **eTable 1.** Number of Participants Who Have EAA Data Available at 1, 2, or All 3 Timepoints (7, 9, and 14 Years) **eTable 2.** Individual and Overlapping Sample Sizes at Three Timepoints (7, 9, and 14 Years) **eTable 3.** Systematic Comparison of Three Available Methods for Calculating Epigenetic Clocks in CHAMACOS Children (Ages 7-14 Years, N = 290) **eTable 4.** Sociodemographic Characteristics of Mother-Child Pairs Included in the Study by Prenatal Maternal Occupation (N = 290) **eTable 5.** Comparison of Sociodemographic Characteristics Between Included and Excluded Mother-Child Pairs **eTable 6.** Adjusted Associations Between Prenatal Maternal Occupation and Child Horvath EAA by Child Age Compared to Children Whose Mothers Did Not Work During Pregnancy (Ages 7-14 Years, N = 290) **eFigure 1.** Performance of Six Epigenetic Clocks in CHAMACOS Children (Ages 7-14 Years, N = 290) **eFigure 2.** Cross-sectional Correlations Between Chronological Age and Epigenetic Age Estimates in CHAMACOS Children **eFigure 3.** Adjusted Associations Between Prenatal Maternal Occupation With Secondary Measures of Child EAA and DNmTLadjAge Compared to Children Whose Mothers Did Not Work During Pregnancy (Ages 7-14 Years, N = 290)
eReferences

Supplement 2.
Data Sharing Statement

## References

[1] Perera F, Herbstman J. Prenatal environmental exposures, epigenetics, and disease. Reprod Toxicol. 2011;31(3):363-373. doi:10.1016/j.reprotox.2010.12.055 21256208 PMC3171169

[2] Zhu Z, Cao F, Li X. Epigenetic programming and fetal metabolic programming. Front Endocrinol (Lausanne). 2019;10:764. doi:10.3389/fendo.2019.00764 31849831 PMC6901800

[3] Teschendorff AE, Menon U, Gentry-Maharaj A, . Age-dependent DNA methylation of genes that are suppressed in stem cells is a hallmark of cancer. Genome Res. 2010;20(4):440-446. doi:10.1101/gr.103606.109 20219944 PMC2847747

[4] Hernandez DG, Nalls MA, Gibbs JR, . Distinct DNA methylation changes highly correlated with chronological age in the human brain. Hum Mol Genet. 2011;20(6):1164-1172. doi:10.1093/hmg/ddq561 21216877 PMC3043665

[5] Perna L, Zhang Y, Mons U, Holleczek B, Saum KU, Brenner H. Epigenetic age acceleration predicts cancer, cardiovascular, and all-cause mortality in a German case cohort. Clin Epigenetics. 2016;8(1):64. doi:10.1186/s13148-016-0228-z 27274774 PMC4891876

[6] Zheng Y, Joyce BT, Colicino E, . Blood epigenetic age may predict cancer incidence and mortality. EBioMedicine. 2016;5:68-73. doi:10.1016/j.ebiom.2016.02.008 27077113 PMC4816845

[7] Chen BH, Marioni RE, Colicino E, . DNA methylation-based measures of biological age: meta-analysis predicting time to death. Aging (Albany NY). 2016;8(9):1844-1865. doi:10.18632/aging.101020 27690265 PMC5076441

[8] Daredia S, Huen K, Van Der Laan L, . Prenatal and birth associations of epigenetic gestational age acceleration in the Center for the Health Assessment of Mothers and Children of Salinas (CHAMACOS) cohort. Epigenetics. 2022;17(13):2006-2021. doi:10.1080/15592294.2022.2102846 35912433 PMC9665122

[9] Bozack AK, Rifas-Shiman SL, Gold DR, . DNA methylation age at birth and childhood: performance of epigenetic clocks and characteristics associated with epigenetic age acceleration in the Project Viva cohort. Clin Epigenetics. 2023;15(1):62. doi:10.1186/s13148-023-01480-2 37046280 PMC10099681

[10] Girchenko P, Lahti J, Czamara D, . Associations between maternal risk factors of adverse pregnancy and birth outcomes and the offspring epigenetic clock of gestational age at birth. Clin Epigenetics. 2017;9:49. doi:10.1186/s13148-017-0349-z 28503212 PMC5422977

[11] Ladd-Acosta C, Vang E, Barrett ES, ; Environmental Influences on Child Health Outcomes Program. Analysis of pregnancy complications and epigenetic gestational age of newborns. JAMA Netw Open. 2023;6(2):e230672. doi:10.1001/jamanetworkopen.2023.0672 36826815 PMC9958528

[12] Dieckmann L, Lahti-Pulkkinen M, Kvist T, . Characteristics of epigenetic aging across gestational and perinatal tissues. Clin Epigenetics. 2021;13(1):97. doi:10.1186/s13148-021-01080-y 33926514 PMC8082803

[13] Clark J, Bulka CM, Martin CL, . Placental epigenetic gestational aging in relation to maternal sociodemographic factors and smoking among infants born extremely preterm: a descriptive study. Epigenetics. 2022;17(13):2389-2403. doi:10.1080/15592294.2022.2125717 36134874 PMC9665142

[14] Song AY, Feinberg JI, Bakulski KM, . Prenatal exposure to ambient air pollution and epigenetic aging at birth in newborns. Front Genet. 2022;13:929416. doi:10.3389/fgene.2022.929416 35836579 PMC9274082

[15] Niemiec SS, Kechris K, Pattee J, . Prenatal exposures to per- and polyfluoroalkyl substances and epigenetic aging in umbilical cord blood: the Healthy Start study. Environ Res. 2023;231(pt 2):116215. doi:10.1016/j.envres.2023.116215 37224946 PMC10330919

[16] Simanek AM, Manansala R, Woo JMP, Meier HCS, Needham BL, Auer PL. Prenatal socioeconomic disadvantage and epigenetic alterations at birth among children born to White British and Pakistani mothers in the Born in Bradford Study. Epigenetics. 2022;17(13):1976-1990. doi:10.1080/15592294.2022.2098569 35837690 PMC9665119

[17] Katrinli S, Smith AK, Drury SS, . Cumulative stress, PTSD, and emotion dysregulation during pregnancy and epigenetic age acceleration in Hispanic mothers and their newborn infants. Epigenetics. 2023;18(1):2231722. doi:10.1080/15592294.2023.2231722 37433036 PMC10337495

[18] de Prado-Bert P, Ruiz-Arenas C, Vives-Usano M, . The early-life exposome and epigenetic age acceleration in children. Environ Int. 2021;155:106683. doi:10.1016/j.envint.2021.106683 34144479

[19] Shiau S, Wang L, Liu H, . Prenatal gestational diabetes mellitus exposure and accelerated offspring DNA methylation age in early childhood. Epigenetics. 2021;16(2):186-195. doi:10.1080/15592294.2020.1790924 32614694 PMC7889277

[20] Khodasevich D, Holland N, Hubbard A, . Associations between prenatal phthalate exposure and childhood epigenetic age acceleration. Environ Res. 2023;231(pt 1):116067. doi:10.1016/j.envres.2023.116067 37149020 PMC10330458

[21] Simpkin AJ, Hemani G, Suderman M, . Prenatal and early life influences on epigenetic age in children: a study of mother-offspring pairs from two cohort studies. Hum Mol Genet. 2016;25(1):191-201. doi:10.1093/hmg/ddv456 26546615 PMC4690495

[22] McGill MG, Pokhvisneva I, Clappison AS, . Maternal prenatal anxiety and the fetal origins of epigenetic aging. Biol Psychiatry. 2022;91(3):303-312. doi:10.1016/j.biopsych.2021.07.025 34756561

[23] Laubach ZM, Bozack A, Aris IM, . Maternal prenatal social experiences and offspring epigenetic age acceleration from birth to mid-childhood. Ann Epidemiol. 2024;90:28-34. doi:10.1016/j.annepidem.2023.10.003 37839726 PMC10842218

[24] Oberlander TF, Weinberg J, Papsdorf M, Grunau R, Misri S, Devlin AM. Prenatal exposure to maternal depression, neonatal methylation of human glucocorticoid receptor gene (NR3C1) and infant cortisol stress responses. Epigenetics. 2008;3(2):97-106. doi:10.4161/epi.3.2.6034 18536531

[25] Braithwaite EC, Kundakovic M, Ramchandani PG, Murphy SE, Champagne FA. Maternal prenatal depressive symptoms predict infant NR3C1 1F and BDNF IV DNA methylation. Epigenetics. 2015;10(5):408-417. doi:10.1080/15592294.2015.1039221 25875334 PMC4622733

[26] Liu Y, Murphy SK, Murtha AP, . Depression in pregnancy, infant birth weight and DNA methylation of imprint regulatory elements. Epigenetics. 2012;7(7):735-746. doi:10.4161/epi.20734 22677950 PMC3414394

[27] Dieckmann L, Czamara D. Epigenetics of prenatal stress in humans: the current research landscape. Clin Epigenetics. 2024;16(1):20. doi:10.1186/s13148-024-01635-9 38308342 PMC10837967

[28] Thomson K, Moffat M, Arisa O, . Socioeconomic inequalities and adverse pregnancy outcomes in the UK and Republic of Ireland: a systematic review and meta-analysis. BMJ Open. 2021;11(3):e042753. doi:10.1136/bmjopen-2020-042753 33722867 PMC7959237

[29] Runkle J, Flocks J, Economos J, Tovar-Aguilar JA, McCauley L. Occupational risks and pregnancy and infant health outcomes in Florida farmworkers. Int J Environ Res Public Health. 2014;11(8):7820-7840. doi:10.3390/ijerph110807820 25101767 PMC4143835

[30] Lima M, Ismail S, Ashworth A, Morris SS. Influence of heavy agricultural work during pregnancy on birthweight in northeast Brazil. Int J Epidemiol. 1999;28(3):469-474. doi:10.1093/ije/28.3.469 10405850

[31] Slesinger DP, Christenson BA, Cautley E. Health and mortality of migrant farm children. Soc Sci Med. 1986;23(1):65-74. doi:10.1016/0277-9536(86)90325-4 3749965

[32] Gwyther ME, Jenkins M. Migrant farmworker children: health status, barriers to care, and nursing innovations in health care delivery. J Pediatr Health Care. 1998;12(2):60-66. doi:10.1016/S0891-5245(98)90223-1 9592438

[33] Hansen E, Donohoe M. Health issues of migrant and seasonal farmworkers. J Health Care Poor Underserved. 2003;14(2):153-164. doi:10.1353/hpu.2010.0790 12739296

[34] Schenker MB, McCurdy SA, Riden HE, Villarejo D. Improving the health of agricultural workers and their families in California. Accessed June 11, 2024. https://ucghi.universityofcalifornia.edu/sites/default/files/ucghi-ag-work-paper-2015.pdf

[35] Eskenazi B, Bradman A, Gladstone EA, Jaramillo S, Birch K, Holland N. CHAMACOS, a longitudinal birth cohort study: lessons from the fields. J Child Health. 2003;1(1):3-27. doi:10.3109/713610244

[36] Eskenazi B, Harley K, Bradman A, . Association of in utero organophosphate pesticide exposure and fetal growth and length of gestation in an agricultural population. Environ Health Perspect. 2004;112(10):1116-1124. doi:10.1289/ehp.6789 15238287 PMC1247387

[37] Sandoval J, Heyn H, Moran S, . Validation of a DNA methylation microarray for 450,000 CpG sites in the human genome. Epigenetics. 2011;6(6):692-702. doi:10.4161/epi.6.6.16196 21593595

[38] Pidsley R, Zotenko E, Peters TJ, . Critical evaluation of the Illumina MethylationEPIC BeadChip microarray for whole-genome DNA methylation profiling. Genome Biol. 2016;17(1):208. doi:10.1186/s13059-016-1066-1 27717381 PMC5055731

[39] Horvath S. DNA methylation age of human tissues and cell types. Genome Biol. 2013;14(10):R115. doi:10.1186/gb-2013-14-10-r115 24138928 PMC4015143

[40] Horvath S, Oshima J, Martin GM, . Epigenetic clock for skin and blood cells applied to Hutchinson Gilford Progeria Syndrome and *ex vivo* studies. Aging (Albany NY). 2018;10(7):1758-1775. doi:10.18632/aging.101508 30048243 PMC6075434

[41] Hannum G, Guinney J, Zhao L, . Genome-wide methylation profiles reveal quantitative views of human aging rates. Mol Cell. 2013;49(2):359-367. doi:10.1016/j.molcel.2012.10.016 23177740 PMC3780611

[42] Levine ME, Lu AT, Quach A, . An epigenetic biomarker of aging for lifespan and healthspan. Aging (Albany NY). 2018;10(4):573-591. doi:10.18632/aging.101414 29676998 PMC5940111

[43] Lu AT, Seeboth A, Tsai PC, . DNA methylation-based estimator of telomere length. Aging (Albany NY). 2019;11(16):5895-5923. doi:10.18632/aging.102173 31422385 PMC6738410

[44] Lu AT, Quach A, Wilson JG, . DNA methylation GrimAge strongly predicts lifespan and healthspan. Aging (Albany NY). 2019;11(2):303-327. doi:10.18632/aging.101684 30669119 PMC6366976

[45] Thrush KL, Higgins-Chen AT, Liu Z, Levine ME. R methylCIPHER: a methylation clock investigational package for hypothesis-driven evaluation & research. *bioRxiv*. Preprint posted online July 16, 2022. doi:10.1101/2022.07.13.499978

[46] Higgins-Chen AT, Thrush KL, Wang Y, . A computational solution for bolstering reliability of epigenetic clocks: implications for clinical trials and longitudinal tracking. Nat Aging. 2022;2(7):644-661. doi:10.1038/s43587-022-00248-2 36277076 PMC9586209

[47] Horvath S, Gurven M, Levine ME, . An epigenetic clock analysis of race/ethnicity, sex, and coronary heart disease. Genome Biol. 2016;17(1):171. doi:10.1186/s13059-016-1030-0 27511193 PMC4980791

[48] Greenland S, Pearl J, Robins JM. Causal diagrams for epidemiologic research. Epidemiology. 1999;10(1):37-48. doi:10.1097/00001648-199901000-00008 9888278

[49] Department of Pesticide Regulation, California Environmental Protection Agency. *Annual Pesticide Use Report*. Dept of Pesticide Regulation, California Environmental Protection Agency; 2007. Accessed November 1, 2023. https://www.cdpr.ca.gov/docs/pur/purmain.htm

[50] Bradman A, Eskenazi B, Barr DB, . Organophosphate urinary metabolite levels during pregnancy and after delivery in women living in an agricultural community. Environ Health Perspect. 2005;113(12):1802-1807. doi:10.1289/ehp.7894 16330368 PMC1314925

[51] Bradman A, Castorina R, Barr DB, . Determinants of organophosphorus pesticide urinary metabolite levels in young children living in an agricultural community. Int J Environ Res Public Health. 2011;8(4):1061-1083. doi:10.3390/ijerph8041061 21695029 PMC3118878

[52] Gunier RB, Ward MH, Airola M, . Determinants of agricultural pesticide concentrations in carpet dust. Environ Health Perspect. 2011;119(7):970-976. doi:10.1289/ehp.1002532 21330232 PMC3222988

[53] Gunier RB, Bradman A, Harley KG, Kogut K, Eskenazi B. Prenatal residential proximity to agricultural pesticide use and IQ in 7-year-old children. Environ Health Perspect. 2017;125(5):057002. doi:10.1289/EHP504 28557711 PMC5644974

[54] Krieger N, Chen JT, Testa C, . Use of correct and incorrect methods of accounting for age in studies of epigenetic accelerated aging: implications and recommendations for best practices. Am J Epidemiol. 2023;192(5):800-811. doi:10.1093/aje/kwad025 36721372 PMC10160768

[55] Dunn EC, Simpkin AJ, Walton E. Statistical and conceptual considerations in socioepigenomics research on childhood adversity and epigenetic aging. JAMA Netw Open. 2023;6(6):e2317958. doi:10.1001/jamanetworkopen.2023.17958 37307003

[56] R: The R Project for Statistical Computing. Accessed November 14, 2023. https://www.r-project.org/

[57] Barker DJ. The fetal and infant origins of adult disease. BMJ. 1990;301(6761):1111. doi:10.1136/bmj.301.6761.1111 2252919 PMC1664286

[58] Gluckman P, Hanson M, eds. Developmental Origins of Health and Disease. Cambridge University Press; 2006. doi:10.1017/CBO9780511544699

[59] Holmes SM. Fresh Fruit, Broken Bodies: Migrant Farmworkers in the United States. University of California Press; 2013. 10.1080/13648470.2014.929091 24986328

[60] Kim NJE, Vásquez VB, Torres E, Nicola RM, Karr C. Breaking the silence: sexual harassment of Mexican women farmworkers. J Agromedicine. 2016;21(2):154-162. doi:10.1080/1059924X.2016.1143903 26797165 PMC5957069

[61] Larsen AE, Gaines SD, Deschênes O. Agricultural pesticide use and adverse birth outcomes in the San Joaquin Valley of California. Nat Commun. 2017;8(1):302. doi:10.1038/s41467-017-00349-2 28851866 PMC5575123

[62] Henriksen TB, Hedegaard M, Secher NJ, Wilcox AJ. Standing at work and preterm delivery. Br J Obstet Gynaecol. 1995;102(3):198-206. doi:10.1111/j.1471-0528.1995.tb09094.x 7794843

[63] Runge SB, Pedersen JK, Svendsen SW, Juhl M, Bonde JP, Nybo Andersen AM. Occupational lifting of heavy loads and preterm birth: a study within the Danish National Birth Cohort. Occup Environ Med. 2013;70(11):782-788. doi:10.1136/oemed-2012-101173 23839660

[64] Flocks J, Vi Thien Mac V, Runkle J, Tovar-Aguilar JA, Economos J, McCauley LA. Female farmworkers’ perceptions of heat-related illness and pregnancy health. J Agromedicine. 2013;18(4):350-358. doi:10.1080/1059924X.2013.826607 24125050 PMC5682625

[65] Ni W, Nikolaou N, Ward-Caviness CK, . Associations between medium- and long-term exposure to air temperature and epigenetic age acceleration. Environ Int. 2023;178:108109. doi:10.1016/j.envint.2023.108109 37517177 PMC10656697

[66] Lind PM, Salihovic S, Lind L. High plasma organochlorine pesticide levels are related to increased biological age as calculated by DNA methylation analysis. Environ Int. 2018;113:109-113. doi:10.1016/j.envint.2018.01.019 29421399

[67] Clausing ES, Binder AM, Non AL. Epigenetic age associates with psychosocial stress and resilience in children of Latinx immigrants. Epigenomics. 2021;13(21):1677-1699. doi:10.2217/epi-2019-0343 33749330

[68] Corchero-Falcón MDR, Gómez-Salgado J, García-Iglesias JJ, Camacho-Vega JC, Fagundo-Rivera J, Carrasco-González AM. Risk factors for working pregnant women and potential adverse consequences of exposure: a systematic review. Int J Public Health. 2023;68:1605655. doi:10.3389/ijph.2023.1605655 36874222 PMC9977819

[69] Simpkin AJ, Howe LD, Tilling K, . The epigenetic clock and physical development during childhood and adolescence: longitudinal analysis from a UK birth cohort. Int J Epidemiol. 2017;46(2):549-558. doi:10.1093/ije/dyw307 28089957 PMC5722033

[70] Binder AM, Corvalan C, Mericq V, . Faster ticking rate of the epigenetic clock is associated with faster pubertal development in girls. Epigenetics. 2018;13(1):85-94. doi:10.1080/15592294.2017.1414127 29235933 PMC5836971

[71] Suarez A, Lahti J, Czamara D, . The epigenetic clock and pubertal, neuroendocrine, psychiatric, and cognitive outcomes in adolescents. Clin Epigenetics. 2018;10(1):96. doi:10.1186/s13148-018-0528-6 30021623 PMC6052515

[72] Prentice P, Viner RM. Pubertal timing and adult obesity and cardiometabolic risk in women and men: a systematic review and meta-analysis. Int J Obes (Lond). 2013;37(8):1036-1043. doi:10.1038/ijo.2012.177 23164700

