# Supplementary material for: Prenatal Maternal Occupation and Child Epigenetic Age Acceleration in an Agricultural Region: NIMHD Social Epigenomics Program
Source: JAMA Netw Open. 2024 Jul 29;7(7):e2421824. doi: 10.1001/jamanetworkopen.2024.21824 (PMC11287394; doi:10.1001/jamanetworkopen.2024.21824)
Supplement: Supplement 1. — eMethods. Supplemental Methods eTable 1. Number of Participants Who Have EAA Data Available at 1, 2, or All 3 Timepoints (7, 9, and 14 Years) eTable 2. Individual and Overlapping Sample Sizes at Three Timepoints (7, 9, and 14 Years) eTable 3. Systematic Comparison of Three Available Methods for Calculating Epigenetic Clocks in CHAMACOS Children (Ages 7-14 Years, N = 290) eTable 4. Sociodemographic Characteristics of Mother-Child Pairs Included in the Study by Prenatal Maternal Occupation (N = 290) eTable 5. Comparison of Sociodemographic Characteristics Between Included and Excluded Mother-Child Pairs eTable 6. Adjusted Associations Between Prenatal Maternal Occupation and Child Horvath EAA by Child Age Compared to Children Whose Mothers Did Not Work During Pregnancy (Ages 7-14 Years, N = 290) eFigure 1. Performance of Six Epigenetic Clocks in CHAMACOS Children (Ages 7-14 Years, N = 290) eFigure 2. Cross-sectional Correlations Between Chronological Age and Epigenetic Age Estimates in CHAMACOS Children eFigure 3. Adjusted Associations Between Prenatal Maternal Occupation With Secondary Measures of Child EAA and DNmTLadjAge Compared to Children Whose Mothers Did Not Work During Pregnancy (Ages 7-14 Years, N = 290) eReferences [file jamanetwopen-e2421824-s001.pdf]

Supplemental Online Content

Daredia S, Bozack AK, Riddell CA, et al. Prenatal maternal occupation and child epigenetic age acceleration in an agricultural region: NIMHD Social Epigenomics Program. *JAMA Netw Open*. 2024;7(7):e2421824. doi:10.1001/jamanetworkopen.2024.21824

eMethods.

eTable 1. Number of participants who have EAA data available at 1, 2, or all 3 timepoints (7, 9, and 14 years).

eTable 2: Individual and overlapping sample sizes at three timepoints (7, 9, and 14 years).

eTable 3. Systematic comparison of three available methods for calculating epigenetic clocks in CHAMACOS children (ages 7-14 years, N=290).

eTable 4: Sociodemographic characteristics of mother-child pairs included in the study by prenatal maternal occupation (N=290).

eTable 5: Comparison of sociodemographic characteristics between included and excluded mother-child pairs.

eTable 6. Adjusted associations between prenatal maternal occupation and child Horvath EAA by child age compared to children whose mothers did not work during pregnancy (ages 7-14 years, N=290).

eFigure 1. Performance of six epigenetic clocks in CHAMACOS children (ages 7-14 years, N=290).

eFigure 2. Cross-sectional correlations between chronological age and epigenetic age estimates in CHAMACOS children.

eFigure 3. Adjusted associations between prenatal maternal occupation with secondary measures of child EAA and DNmTLadjAge compared to children whose mothers did not work during pregnancy (ages 7-14 years, N=290).

eReferences.

This supplemental material has been provided by the authors to give readers additional information about their work.

© 2024 Daredia S et al. *JAMA Network Open.*

eMethods

DNA Methylation (DNAm) Profiling and Quality Control

Blood samples were refrigerated and transported to the University of California, Berkeley biorepository where samples without anticoagulant were separated into serum and clot and stored at -80° C until analysis. DNA was extracted from child blood samples using QIAamp DNA Blood Maxi Kits (Qiagen, Valencia, CA), as previously described.[1] DNA aliquots of 1 µg were bisulfite converted using Zymo Bisulfite Conversion Kits (Zymo Research, Orange, CA). DNA was amplified, enzymatically fragmented, purified, and applied to the Illumina Infinium HumanMethylation450 (450K) BeadChip for age 9 samples and EPIC BeadChip for ages 7 and 14 samples, according to the Illumina protocol (Illumina, San Diego, CA) to measure DNA methylation.[2,3]

Quality control steps included the use of repeats and randomization of samples across chips and plates.[4] Methylation data were imported into R statistical software for preprocessing using the *minifi* package.[5] Quality control was performed at the sample level, excluding samples with overall low intensities (< 10.5) and technical duplicates. We computed detection P values relative to control probes and excluded probes with non-significant detection (P > 0.01) for 5% or more of the samples. Data were preprocessed using functional normalization[6] and adjusted for probe-type bias using the regression on correlated probes method.[7] CombBat from the *sva* package was used to adjust for sample plate as a technical batch.[8] Data were visualized using density distributions at all processing steps and PC analyses were performed to examine the associations of methylation differences with technical, biological, and measured traits with global DNAm variation using PCA plots.

Intrinsic Epigenetic Age Acceleration

Estimated proportions of blood CD8 T cells, CD4 T cells, natural killer cells, B cells, monocytes, and granulocytes were generated by the Clock Foundation calculator[9] using the Houseman algorithm.[10] The calculator also estimated intrinsic epigenetic age acceleration (IEAA), a residual value calculated by regressing Horvath epigenetic age on chronological age and adjusting for the estimated blood cell counts which are known to change with age.

© 2024 Daredia S et al. *JAMA Network Open.*

eTable 1. Number of participants who have EAA data available at 1, 2, or all 3 timepoints (7, 9, and 14 years)

Number of timepoints	Number of participants	Percentage of participants
1	92	31.7
2	80	27.6
3	118	40.7
Total	290	100.0

EAA, Epigenetic Age Acceleration.

eTable 2: Individual and overlapping sample sizes at three timepoints (7, 9, and 14 years)

	Age 7	Age 9	Age 14
**Age 7**	182	143	134
**Age 9**	-	239	157
**Age 14**	-	-	185

© 2024 Daredia S et al. *JAMA Network Open.*

eTable 3. Systematic comparison of three available methods for calculating epigenetic clocks in CHAMACOS children (ages 7-14 years, N=290)

	**Method 1: methylCIPHER *R* Package[1]**			**Method 2: Clock Foundation Online Calculator[2]**			**Method 3: Principal Component-Based Estimation[3,4]**		
**Clock**	*r* (95% CI)	p-value	MAE	*r* (95% CI)	p-value	MAE	*r* (95% CI)	p-value	MAE
Horvath	0.84 (0.82, 0.86)	$< 2.2\times10^{-16}$	2.1	0.62 (0.57, 0.66)	$< 2.2\times10^{-16}$	2.5	**0.84 (0.81, 0.86)**	**$< 2.2\times10^{-16}$**	**1.5**
Skin & Blood	0.73 (0.69, 0.76)	$< 2.2\times10^{-16}$	2.5	**0.92 (0.90, 0.93)**	**$< 2.2\times10^{-16}$**	**2.0**	0.81 (0.78, 0.83)	$< 2.2\times10^{-16}$	2.6
Hannum	0.33 (0.26, 0.40)	$< 2.2\times10^{-16}$	6.5	0.33 (0.26, 0.40)	$< 2.2\times10^{-16}$	6.5	**0.78 (0.74, 0.81)**	**$< 2.2\times10^{-16}$**	**4.1**
PhenoAge	**0.78 (0.75, 0.81)**	**$< 2.2\times10^{-16}$**	**4.2**	0.70 (0.66, 0.74)	$< 2.2\times10^{-16}$	14.3	0.72 (0.68, 0.76)	$< 2.2\times10^{-16}$	14.7
DNAmTL[a]	-0.57 (-0.62, -0.51)	$< 2.2\times10^{-16}$	-	-0.60 (-0.65, -0.54)	$< 2.2\times10^{-16}$	-	**-0.66 (-0.70, -0.61)**	**$< 2.2\times10^{-16}$**	-
GrimAge[b]	-	-	-	**0.76 (0.72, 0.79)**	**$< 2.2\times10^{-16}$**	**5.6**	0.74 (0.70, 0.77)	$< 2.2\times10^{-16}$	15.9

CI, confidence interval; MAE, median absolute error.

Correlation coefficients *r* and 95% CIs from Pearson correlations between chronological age and estimated epigenetic age.
MAE calculated as the median of the absolute difference between estimated epigenetic age and chronological age. A lower MAE indicates that epigenetic age is a better predictor of chronological age.
Bold: Epigenetic aging measure selected for statistical analyses based on highest correlation with chronological age followed by lowest MAE.

[a]Since DNAmTL is an estimator of telomere length which decreases with increasing age, the method with the highest negative correlation with chronological age was chosen.
[b]GrimAge estimates are not available with the methylCIPHER R package

© 2024 Daredia S et al. *JAMA Network Open.*

eTable 4. Sociodemographic characteristics of mother-child pairs included in the study by prenatal maternal occupation (N=290). Values represent count (%) or mean (SD).

	Agricultural field work (N=90)	Other agricultural work (N=40)	Non-agricultural work (N=53)	Did not work (N=107)
**Maternal characteristics**				
**Age at delivery, years**	26.8 (5.1)	26.9 (5.5)	25.8 (5.3)	26.4 (5.1)
**Pre-pregnancy BMI, kg/m^2**	26.8 (4.3)	27.4 (5.9)	27.2 (5.0)	28.0 (6.1)
**Highest level of education**				
≤ 6th grade	50 (55.6)	18 (45.0)	11 (20.8)	49 (45.8)
7th-12th grade	30 (33.3)	16 (40.0)	17 (32.1)	39 (36.4)
≥ High school	10 (11.1)	6 (15.0)	25 (47.2)	19 (17.8)
**Marital status**				
Married	42 (46.7)	13 (32.5)	20 (37.7)	58 (54.2)
Living as married	30 (33.3)	23 (57.5)	17 (32.1)	35 (32.7)
Separated	5 (5.6)	0 (0.0)	3 (5.7)	3 (2.8)
Divorced	0 (0.0)	1 (2.5)	1 (1.9)	3 (2.8)
Single	13 (14.4)	3 (7.5)	12 (22.6)	7 (6.5)
Missing	0 (0.0)	0 (0.0)	0 (0.0)	1 (0.9)
**Parity**				
Nulliparous	24 (26.7)	15 (37.5)	21 (39.6)	35 (32.7)
Multiparous	66 (73.3)	25 (62.5)	32 (60.4)	72 (67.3)
**Country of origin**				
USA	1 (1.1)	3 (7.5)	19 (35.8)	10 (9.3)
Mexico	89 (98.9)	37 (92.5)	34 (64.2)	94 (87.9)
Other	0 (0.0)	0 (0.0)	0 (0.0)	3 (2.8)
**Years in USA at child's birth**				
≤ 1 year	18 (20.0)	3 (7.5)	6 (11.3)	25 (23.4)
2-5 years	30 (33.3)	16 (40.0)	10 (18.9)	25 (23.4)
6-10 years	33 (36.7)	13 (32.5)	8 (15.1)	32 (29.9)
≥ 11 years	9 (10.0)	8 (20.0)	12 (22.6)	16 (15.0)
Entire life	0 (0.0)	0 (0.0)	17 (32.1)	9 (8.4)
**Poverty status during pregnancy**				
At or below poverty line	62 (68.9)	21 (52.5)	22 (41.5)	74 (69.2)
Between poverty line and 200%	24 (26.7)	16 (40.0)	30 (56.6)	30 (28.0)
>200% poverty line	4 (4.4)	3 (7.5)	1 (1.9)	3 (2.8)
**Smoking during pregnancy**				
No	88 (97.8)	39 (97.5)	50 (94.3)	102 (95.3)
Yes	2 (2.2)	1 (2.5)	3 (5.7)	5 (4.7)
**Alcohol consumption during pregnancy**				
No	70 (77.8)	28 (70.0)	44 (83.0)	79 (73.8)
Yes	20 (22.2)	11 (27.5)	9 (17.0)	27 (25.2)
Missing	0 (0.0)	1 (2.5)	0 (0.0)	1 (0.9)
**Mean prenatal urinary DAPs, nmol/g creatinine**	253.6 (269.6)	329.7 (412.0)	326.9 (453.0)	281.5 (323.3)
Missing, n	1	0	0	0
**Prenatal wind-weighted kg of OP pesticides applied within 1 km of residence**	22.9 (34.4)	12.0 (15.7)	21.0 (43.3)	26.9 (34.8)
Missing, n	1	0	0	0
**Mother's physical difficulty at the workplace during pregnancy**				
Not at all strenuous	3 (3.3)	7 (17.5)	20 (37.7)	0 (0.0)
Not very strenuous	17 (18.9)	7 (17.5)	10 (18.9)	0 (0.0)
Somewhat strenuous	37 (41.1)	12 (30.0)	14 (26.4)	0 (0.0)

© 2024 Daredia S et al. *JAMA Network Open.*

Very strenuous	28 (31.1)	6 (15.0)	4 (7.5)	0 (0.0)
Not applicable	5 (5.6)	7 (17.5)	5 (9.4)	107 (100.0)
Missing	0 (0.0)	1 (2.5)	0 (0.0)	0 (0.0)
**Mean mothers' hours per day standing on feet at workplace during pregnancy**	2.7 (2.4)	5.0 (3.0)	3.8 (2.7)	N/A
**Mean mothers' hours per day stooping or bending at workplace during pregnancy**	1.7 (2.1)	0.6 (1.2)	0.8 (1.3)	N/A
**Child characteristics**				
**Sex**				
Female	45 (50.0)	26 (65.0)	25 (47.2)	56 (52.3)
Male	45 (50.0)	14 (35.0)	28 (52.8)	51 (47.7)

BMI, body mass index; DAP, dialkylphosphate; OP, organophosphate.

© 2024 Daredia S et al. *JAMA Network Open.*

eTable 5. Comparison of sociodemographic characteristics between included and excluded mother-child pairs. Values represent count (%) or mean (SD).

	Initial CHAMACOS cohort enrollees (N=601)[a]	Mother-child pairs included in analyses (N=290)	Mother-child pairs excluded from analyses (N=316)
**Maternal characteristics**			
**Age at delivery, years**	26.0 (5.2)	26.5 (5.2)	25.3 (5.1)
Missing, n	62	0	62
**Pre-pregnancy BMI, kg/m^2**	27.0 (5.2)	27.4 (5.4)	26.6 (4.9)
Missing, n	63	0	63
**Highest level of education**			
≤ 6th grade	261 (43.4)	128 (44.1)	136 (43.0)
7th-12th grade	219 (36.4)	102 (35.2)	118 (37.3)
≥ High school	120 (20.0)	60 (20.7)	61 (19.3)
Missing	1 (0.2)	0 (0.0)	1 (0.3)
**Marital status**			
Married	271 (45.1)	133 (45.9)	140 (44.3)
Living as married	210 (34.9)	105 (36.2)	106 (33.5)
Separated	26 (4.3)	11 (3.8)	15 (4.7)
Divorced	6 (1.0)	5 (1.7)	1 (0.3)
Single	86 (14.3)	35 (12.1)	52 (16.5)
Missing	2 (0.3)	1 (0.3)	2 (0.6)
**Parity**			
Nulliparous	211 (35.1)	95 (32.8)	118 (37.3)
Multiparous	388 (64.6)	195 (67.2)	196 (62.0)
Missing	2 (0.3)	0 (0.0)	2 (0.6)
**Country of origin**			
USA	77 (12.8)	33 (11.4)	44 (13.9)
Mexico	509 (84.7)	254 (87.6)	260 (82.3)
Other	14 (2.3)	3 (1.0)	11 (3.5)
Missing	1 (0.2)	0 (0.0)	1 (0.3)
**Years in USA at child's birth**			
≤ 1 year	123 (20.5)	52 (17.9)	71 (22.5)
2-5 years	174 (29.0)	81 (27.9)	95 (30.1)
6-10 years	140 (23.3)	86 (29.7)	55 (17.4)
≥ 11 years	98 (16.3)	45 (15.5)	55 (17.4)
Entire life	65 (10.8)	26 (9.0)	39 (12.3)
Missing	1 (0.2)	0 (0.0)	1 (0.3)
**Poverty status during pregnancy**			
At or below poverty line	369 (61.4)	179 (61.7)	193 (61.1)
Between poverty line and 200%	208 (34.6)	100 (34.5)	110 (34.8)
>200% poverty line	22 (3.7)	11 (3.8)	11 (3.5)
Missing	2 (0.3)	0 (0.0)	2 (0.6)
**Smoking during pregnancy**			
No	565 (94.0)	279 (96.2)	291 (92.1)
Yes	35 (5.8)	11 (3.8)	24 (7.6)
Missing	1 (0.2)	0 (0.0)	1 (0.3)
**Alcohol consumption during pregnancy**			
No	390 (64.9)	221 (76.2)	172 (54.4)
Yes	135 (22.5)	67 (23.1)	70 (22.2)
Missing	76 (12.6)	2 (0.7)	74 (23.4)
**Occupation during pregnancy**			
Agricultural field work	162 (27.0)	90 (31.0)	73 (23.1)

© 2024 Daredia S et al. *JAMA Network Open.*

Other agricultural work	80 (13.3)	40 (13.8)	40 (12.7)
Non-agricultural work	125 (20.8)	53 (18.3)	73 (23.1)
Did not work	189 (31.4)	107 (36.9)	85 (26.9)
Missing	45 (7.5)	0 (0.0)	45 (14.2)
**Mean prenatal urinary DAPs, nmol/g creatinine**	420.7 (2304.9)	287.9 (348.7)	542.6 (3166.1)
Missing, n	5	1	4
**Prenatal wind-weighted kg of OP pesticides applied within 1 km of residence**	21.7 (32.5)	22.5 (34.7)	21.0 (29.5)
Missing, n	70	1	69
**Child characteristics**			
**Sex**			
Female	274 (45.2)	152 (52.4)	122 (38.6)
Male	271 (44.7)	138 (47.6)	133 (42.1)
Missing	61 (10.1)	0 (0.0)	61 (19.3)

BMI, body mass index; DAP, dialkylphosphate; OP, organophosphate.

[a]Five pregnant participants who were enrolled in the initial CHAMACOS cohort eventually delivered twins.

eTable 6. Adjusted associations between prenatal maternal occupation and child Horvath EAA by child age compared to children whose mothers did not work during pregnancy (ages 7-14 years, N=290)

	β	95% CI	p-value
Intercept	-1.83	(-3.72, 0.06)	0.06
Child age (centered)	-0.07	(-0.16, 0.02)	0.14
Prenatal maternal agricultural field work	0.38	(-0.17, 0.92)	0.17
Prenatal maternal other agricultural work	0.10	(-0.65, 0.85)	0.79
Prenatal maternal non-agricultural work	-0.29	(-0.96, 0.39)	0.40
Child age (centered) × prenatal maternal agricultural field work	0.16	(0.02, 0.29)	0.02
Child age (centered) × prenatal maternal other agricultural work	0.00	(-0.17, 0.18)	0.98
Child age (centered) × prenatal maternal non-agricultural work	0.08	(-0.08, 0.24)	0.32

EAA, Epigenetic Age Acceleration; CI, confidence interval; DAP, dialkylphosphate.

Regression coefficients in years and 95% CIs derived from linear mixed effects models with additional statistical interaction term between child age and prenatal maternal occupation.

Adjusted for sociodemographic covariates (maternal age at delivery, pre-pregnancy BMI, baseline maternal education, baseline maternal marital status, parity, poverty status during pregnancy, smoking and alcohol consumption during pregnancy, and child sex) and prenatal OP pesticide exposure (log_{10}-transformed mean prenatal urinary DAP concentrations and log_2-transformed kilograms of OP pesticides used within 1 kilometer of maternal residence during pregnancy).

© 2024 Daredia S et al. *JAMA Network Open.*

<u>eFigure 1</u>. Performance of six epigenetic clocks in CHAMACOS children (ages 7-14 years, N=290)

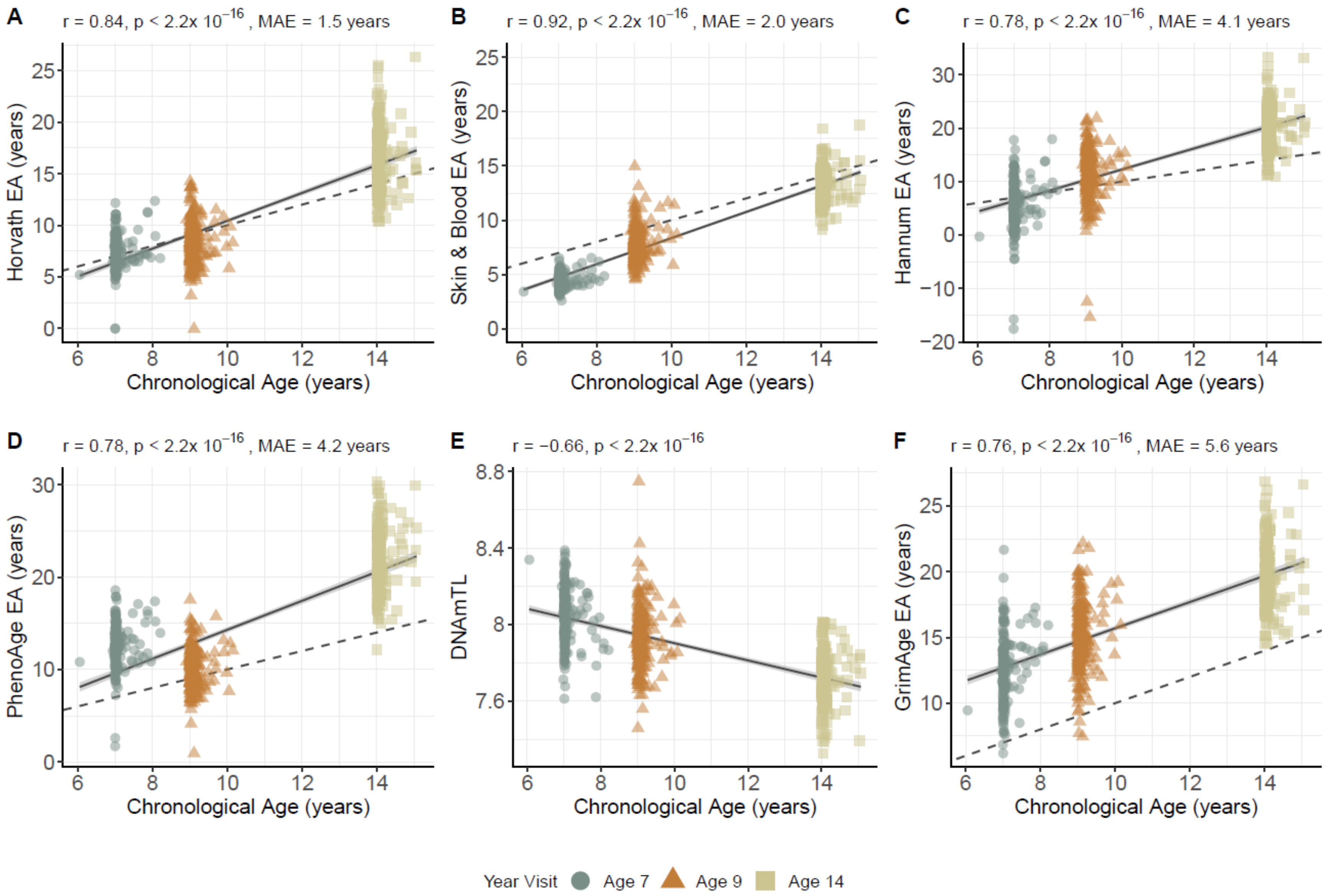

Pearson correlation coefficient *r* and median absolute error (MAE) between child chronological age based on birth date and epigenetic age (EA) estimated by the (A) Horvath Pan-Tissue, (B) Skin & Blood, (C) Hannum, (D) PhenoAge, (E) DNAmTL, and (F) GrimAge epigenetic clocks. The linear trendline and 95% CI are plotted as a solid line with shaded area and the identity line (y = x) is plotted as a dashed line.

© 2024 Daredia S et al. *JAMA Network Open.*

eFigure 2. **Cross-sectional correlations between chronological age and epigenetic age in CHAMACOS children**

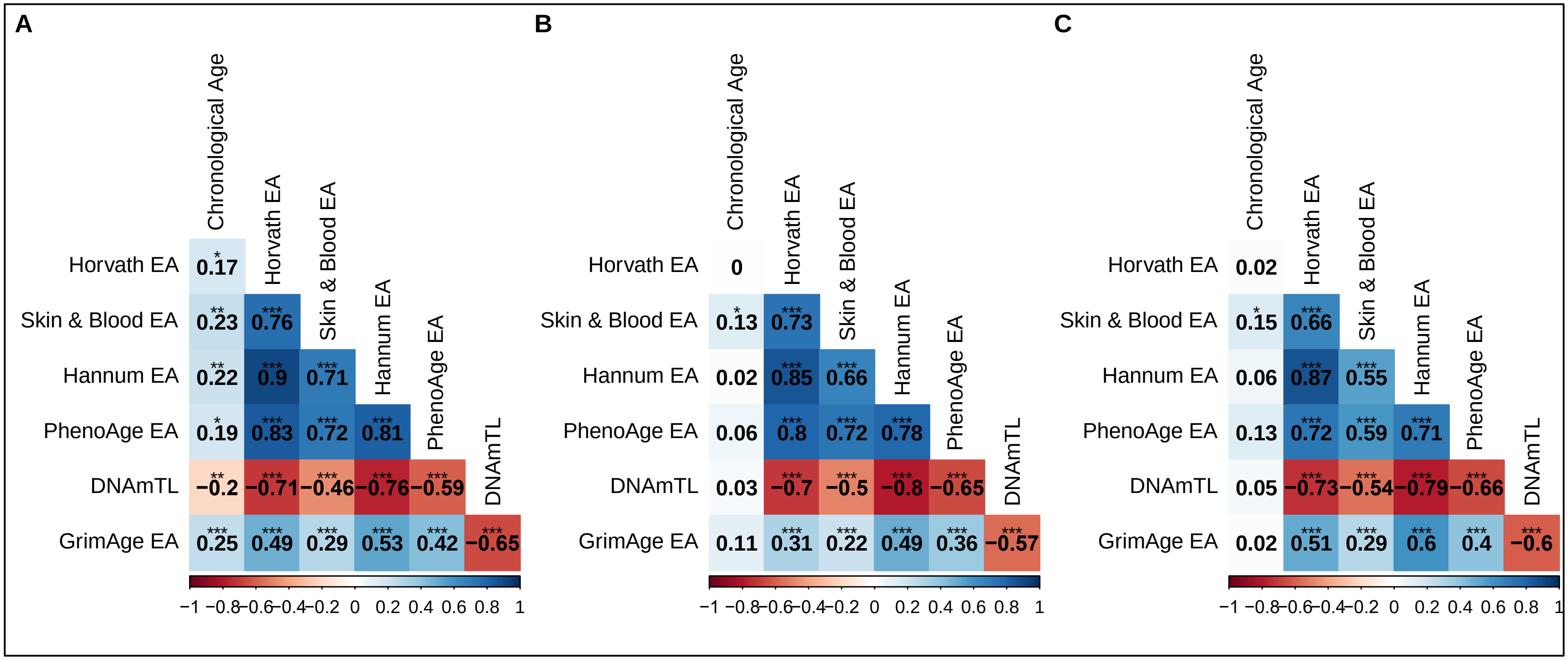

Pearson correlation coefficients *r* between child chronological age and epigenetic age (EA) estimates at **(A)** age 7 years (N=182), **(B)** age 9 years (N=239), and **(C)** age 14 years (N=185). **p*<0.05; ***p*<0.01; ****p*<0.001.

© 2024 Daredia S et al. *JAMA Network Open.*

<u>eFigure 3</u>. Adjusted associations between prenatal maternal occupation with secondary measures of child EAA and DNmTLadjAge compared to children whose mothers did not work during pregnancy (ages 7-14 years, N=290)

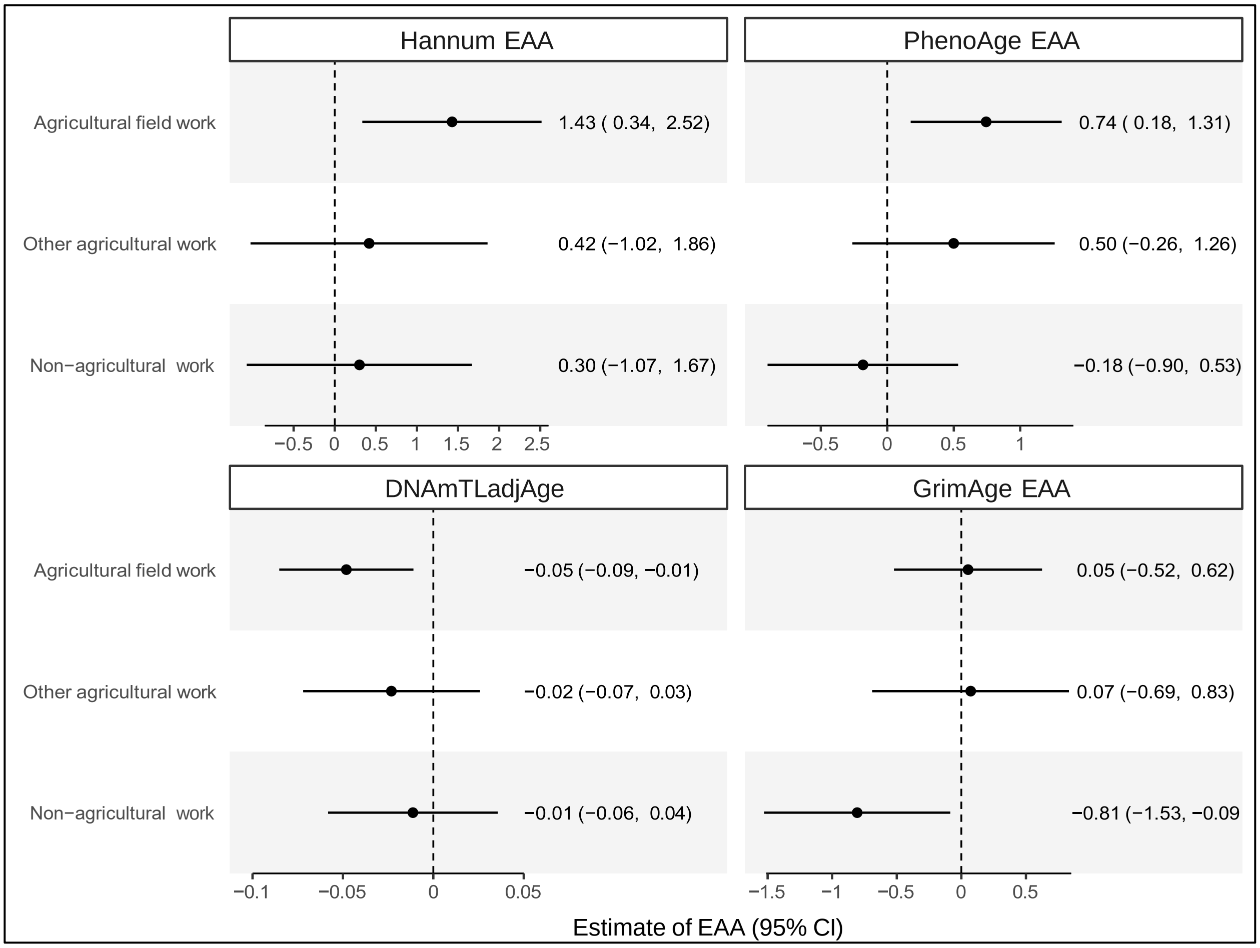

EAA, Epigenetic Age Acceleration; CI, confidence interval; DAP, dialkylphosphate.

Regression coefficients in years and 95% CIs derived from linear mixed effects models adjusted for sociodemographic covariates (maternal age at delivery, pre-pregnancy BMI, baseline maternal education, baseline maternal marital status, parity, poverty status during pregnancy, smoking and alcohol consumption during pregnancy, and child sex) and prenatal OP pesticide exposure (log_{10}-transformed mean prenatal urinary DAP concentrations and log_2-transformed kilograms of OP pesticides used within 1 kilometer of maternal residence during pregnancy).

<u>Outcomes</u>: Residuals from models regressing Hannum, PhenoAge, and GrimAge EA on chronological age represent epigenetic age acceleration (years). Residuals from models regressing DNAmTL on chronological age represent an age-adjusted estimate of DNAmTL (referred to as DNAmTLadjAge) measured in kilobases.

© 2024 Daredia S et al. *JAMA Network Open.*

eReferences

1. Holland N, Furlong C, Bastaki M, et al. Paraoxonase Polymorphisms, Haplotypes, and Enzyme Activity in Latino Mothers and Newborns. *Environ Health Perspect*. 2006;114(7):985-991. doi:10.1289/ehp.8540
2. Sandoval J, Heyn H, Moran S, et al. Validation of a DNA methylation microarray for 450,000 CpG sites in the human genome. *Epigenetics*. 2011;6(6):692-702. doi:10.4161/epi.6.6.16196
3. Pidsley R, Zotenko E, Peters TJ, et al. Critical evaluation of the Illumina MethylationEPIC BeadChip microarray for whole-genome DNA methylation profiling. *Genome Biology*. 2016;17(1):208. doi:10.1186/s13059-016-1066-1
4. Yousefi P, Huen K, Davé V, Barcellos L, Eskenazi B, Holland N. Sex differences in DNA methylation assessed by 450 K BeadChip in newborns. *BMC Genomics*. 2015;16:911. doi:10.1186/s12864-015-2034-y
5. Aryee MJ, Jaffe AE, Corrada-Bravo H, et al. Minfi: a flexible and comprehensive Bioconductor package for the analysis of Infinium DNA methylation microarrays. *Bioinformatics*. 2014;30(10):1363-1369. doi:10.1093/bioinformatics/btu049
6. Fortin JP, Labbe A, Lemire M, et al. Functional normalization of 450k methylation array data improves replication in large cancer studies. *Genome Biology*. 2014;15(11):503. doi:10.1186/s13059-014-0503-2
7. Niu L, Xu Z, Taylor JA. RCP: a novel probe design bias correction method for Illumina Methylation BeadChip. *Bioinformatics*. 2016;32(17):2659-2663. doi:10.1093/bioinformatics/btw285
8. Johnson WE, Li C, Rabinovic A. Adjusting batch effects in microarray expression data using empirical Bayes methods. *Biostatistics*. 2007;8(1):118-127. doi:10.1093/biostatistics/kxj037
9. Horvath S. DNA methylation age of human tissues and cell types. *Genome Biology*. 2013;14(10):3156. doi:10.1186/gb-2013-14-10-r115
10. Houseman EA, Accomando WP, Koestler DC, et al. DNA methylation arrays as surrogate measures of cell mixture distribution. *BMC Bioinformatics*. 2012;13(1):86. doi:10.1186/1471-2105-13-86
11. MorganLevineLab. MorganLevineLab/methylCIPHER. Accessed September 1, 2023. https://github.com/MorganLevineLab/methylCIPHER
12. DNA Methylation Age Calculator | DNAm Age Calculator, powered by Clock Foundation. Accessed September 6, 2023. https://dnamage.clockfoundation.org/
13. Albert T. HC, Kyra L. T, Yunzhang W, et al. A computational solution for bolstering reliability of epigenetic clocks: Implications for clinical trials and longitudinal tracking. Accessed October 3, 2023. https://github.com/MorganLevineLab/PC-Clocks
14. Higgins-Chen AT, Thrush KL, Wang Y, et al. A computational solution for bolstering reliability of epigenetic clocks: Implications for clinical trials and longitudinal tracking. *Nat Aging*. 2022;2(7):644-661. doi:10.1038/s43587-022-00248-2

© 2024 Daredia S et al. *JAMA Network Open.*
