# Supplementary material for: Prenatal Maternal Occupation and Child Epigenetic Age Acceleration in an Agricultural Region: NIMHD Social Epigenomics Program
Source: JAMA Netw Open. 2024 Jul 29;7(7):e2421824. doi: 10.1001/jamanetworkopen.2024.21824 (PMC11287394; doi:10.1001/jamanetworkopen.2024.21824)
Supplement: Supplement 2. — Data Sharing Statement [file jamanetwopen-e2421824-s002.pdf]

Data Sharing Statement

Daredia. Prenatal Maternal Occupation and Child Epigenetic Age Acceleration in an Agricultural Region. *JAMA Netw Open*. Published July 29, 2024.
doi:10.1001/jamanetworkopen.2024.21824

Data
Data available: No

Additional Information
Explanation for why data not available: Datasets generated and analyzed during the current study are available from the corresponding authors with appropriate permission from the CHAMACOS study team and investigators upon reasonable request and institutional review board approval.
